# Efficiency of hCG for Inducing Resumption of Ovarian Cyclicity and Synchronized Ovulations during the Seasonal Anestrous in Sheep

**DOI:** 10.3390/ani11113159

**Published:** 2021-11-05

**Authors:** Zurisaday Santos-Jimenez, César A. Meza-Herrera, Guadalupe Calderon-Leyva, Paula Martinez-Ros, Juan M. Guillen-Muñoz, Antonio Gonzalez-Bulnes

**Affiliations:** 1 Unidad Laguna, Universidad Autónoma Agraria Antonio Narro, Torreón 25315, Coahuila, Mexico; mvz_zusan@hotmail.com (Z.S.-J.); gcalderon06@hotmail.com (G.C.-L.); 2 Departamento de Farmacologia y Toxicologia, Facultad de Veterinaria, UCM, Ciudad Universitaria s/n, 28040 Madrid, Spain; 3 Unidad Regional Universitaria de Zonas Áridas, Universidad Autónoma Chapingo, Unidad Regional Universitaria de Zonas Áridas, Bermejillo 35230, Durango, Mexico; cmeza2020@hotmail.com; 4 Departamento de Produccion y Sanidad Animal, Facultad de Veterinaria, Universidad Cardenal Herrera-CEU, CEU Universities, C/Tirant lo Blanc 7, Alfara del Patriarca, 46115 Valencia, Spain; paula.martinez@uchceu.es

**Keywords:** anestrous sheep, induction estrus, hCG, induction ovulation

## Abstract

**Simple Summary:**

The use of equine chorionic gonadotrophin (eCG) in protocols for estrus synchronization in sheep is currently challenged, so it is necessary to implement alternatives (i.e., human chorionic gonadotropin; hCG), mainly during the anestrous season. Therefore, we compared the reproductive outcomes, including estrus induction, ovulatory follicle dynamics, and pregnancy outputs in anestrus Dorper sheep treated with insertion of one intravaginal progesterone-loaded CIDR + either saline, eCG, or hCG. The administration of hCG at the time of CIDR removal was effective at inducing estrus, resume ovulations, and ovarian cyclicity, yet, in a narrow window of time, it is a scenario that may limit the implementation of an artificial insemination protocol.

**Abstract:**

This research aimed to evaluate whether the administration of hCG at the time of removal of a progesterone device may be effective at inducing estrus and ovulations in sheep during the natural seasonal anestrous, by comparing reproductive outputs (induction and duration of estrus, follicle development, ovulation, ovulation rate, and pregnancy rates) in ewes treated with eCG or only saline solution at the time of CIDR removal. Whereas results demonstrated no response in the control non-treated group, the largest rate of narrowly synchronized estrus signs and ovulations occurred in the eCG-group. The administration of hCG was effective at inducing estrus, promote follicular growth and a delayed yet significant ovulation (>84%) on day 10 after CIDR removal; moreover, an increased embryonic implantation rate was also observed. Moreover, if the hCG ewes remain exposed to active males for some days, said strategy could be adopted, aiming to induce pregnancies by natural mating in a short period of time.

## 1. Introduction

The reproductive management of sheep, during both the reproductive and non-reproductive seasons, mainly relies on the use of progesterone-based protocols combined with the administration of a single dose of equine chorionic gonadotrophin (eCG) at the end of the progesterone treatment [1], which induces the ovulation and allows its synchronization. Accordingly, it is essential when implementing artificial insemination, but it is also of high interest for increasing the number of twin births [2]. However, the use of eCG is currently challenged by a strong animal rights movement against obtaining eCG from pregnant mares, which makes the banning of this hormone foreseeable [3,4]. Currently, diverse laboratories around the world are developing a synthetic eCG by recombinant deoxyribonucleic acid (DNA) technology, which may function in a similar way as follicle stimulating hormone (FSH) and luteinizing hormone (LH) [5,6,7,8,9]. However, these efforts are still preliminary, and results are far away of being translated to practice.

Unavailability of eCG is a critical problem for the management of reproduction in farm animals since the different attempts of using other hormones, such as FSH, LH, gonadotrophin-releasing hormone (GnRH), or hCG have not found similar outcomes as eCG [4]. There are promising results applying GnRH in saline at 56 h of progesterone withdrawal [9] or applying the hormone at 36 h if using a slow-release vehicle [10], but the yields seem to be weakened during the non-reproductive season [11]. The characteristics of hCG, mainly its high similarity to LH and affinity for the same receptors [12,13], favored its use for inducing ovulations when applying protocols for estrus synchronization in sheep [14,15]. Yet, recent studies have evidenced a decrease in fertility due to deleterious effects on the development of ovulatory follicles [16,17]. However, these studies were performed during reproductive season and the differences in the follicle dynamics occurring during the seasonal anestrous [18], due to the different patterns of release of LH [19], are currently well known.

We hypothesized that administration of an acute discharge of a hormone with LH-like activity, such as hCG, may induce the resumption of the ovulatory activity and the synchronization of ovulations in sheep during seasonal anestrous. Therefore, we compared the reproductive behavior, ovulatory follicle dynamics, and pregnancy outputs of Dorper sheep treated with progesteroneimpregnated CIDR, plus either saline, eCG, or hCG during the seasonal anestrous.

## 2. Materials and Methods

### 2.1. Ethics Statement

The present study was carried out, under conditions of natural photoperiod during the non-breeding season (April), in a single commercial farm in the north of Mexico (Ejido Granada, Matamoros, Coahuila de Zaragoza, Mexico, latitude of 25.3 N, altitude of 1115 m.a.s.l.). The research was performed according to the international [20] and national [21] guidelines for the ethical care and protection of animals used for research. All of the methods and management procedures in this study were evaluated and approved by the Institutional Committee of Ethics in Animal Research of the Universidad Autónoma Agraria Antonio Narro (approval reference number UAAAN/UL/1330-8241-2868).

### 2.2. Animals and Experimental Procedure

The trial involved multiparous Dorper ewes (*n* = 36) in adequate health status, with an average mean live weight (LW, 43.7 ± 1.4 kg) and body condition score (BCS, 2.5; scale of 0–5, being 0 = very thin and 5 = very fat). Seasonal anestrous was confirmed by an ovarian ultrasonography for determining absence/presence of corpora lutea with a 7.5 MHz transrectal ultrasound (Aloka SSD 500, Aloka Co. Ltd., Tokyo, Japan). All of the animals were treated with one intravaginal CIDR device (CIDR^®^ Ovis, Zoetis, Cd. de Mexico, Mexico) for seven days plus one intramuscular dose of 5 mg of prostaglandin F_2α_ (Lutalyse, Zoetis, Cd. de Mexico, Mexico) at CIDR withdrawal. The group of females remained together, but for experimental purposes, they were divided into three experimental subgroups based on the gonadotrophin treatment at CIDR removal. The first group (Group eCG, *n* = 11) received one intramuscular injection of 300 IU of equine chorionic gonadotrophin (GonActive^®^ eCG, Virbac, Zapopan, Mexico), the second group (Group hCG, *n* = 13) received one intramuscular injection of 300 IU of human chorionic gonadotrophin (Chorulon^®^, MSD, Cd. de Mexico, Mexico), while the third group received one intramuscular injection of saline solution and acted as the control group (Group CON, *n* = 12). The response variables evaluated were the occurrence and timing of estrus behavior, ovarian follicle dynamics, occurrence, and timing of ovulation/luteinization of preovulatory follicles, and the presence and number of corpora lutea in response to the treatment and pregnancy rate.

### 2.3. Onset and Duration of Estrus Behavior

Occurrence of estrus behavior was determined with trained rams every 12 h from CIDR withdrawal. Each sheep was exposed to males until ewes showed no more signs of estrus (refused the contact with the male) or until 72 h after CIDR removal, in case they did not exhibited estrus behavior. Mating was allowed for determining fertility and pregnancy rates.

### 2.4. Ovarian Follicle Dynamics, Occurrence of Ovulation, and Pregnancy Diagnosis

Ultrasonographic assessment of follicle dynamics was performed in all of the ewes showing estrus signs, every 24 h after CIDR removal. All follicles with ≥4 mm in size were assessed by means of 7.5 MHz transrectal ultrasound (Aloka SSD 500). Ovulation was determined by evaluating the disappearance of the ovulatory follicles recorded in a previous ultrasound scanning, as formerly described [22]. Ultrasonography was subsequently performed to determine the presence and number of corpora lutea and/or anovulatory follicles (i.e., day 10), as well as pregnancy rate, considering both the presence and number of embryos (i.e., day 32) after CIDR removal.

### 2.5. Statistical Analyses

The effects of the treatment on the occurrence and timing of estrus and ovulation, ovarian follicle dynamics, number of corpora lutea as well as pregnancy and twinning rates were assessed by means of analysis of variance (ANOVA) and chi-square test using SPSS 22.0 (IBM Corporation, New York, NY, USA). The statistical analysis of the results expressed as percentages was performed after the arcsine transformation of the values for each individual percentage, after the data normality test. All results in the main text and tables are expressed as mean ± S.E.M. and statistical significance was accepted at *p* < 0.05.

## 3. Results

The assessment of the reproductive response in all of the sheep showed that the CIDR treatment during seasonal anestrous in absence of any hormone for inducing or mimicking LH secretion (Group CON) was unable to trigger estrus induction and, therefore, neither ovulations nor pregnancies were observed during the experimental period (Table 1). Interestingly, however, control ewes were able to display a subsequent resumption of ovarian cyclic activity and ovulations in around half of the ewes, as indicated when evaluating presence or absence of corpora lutea on day 10 after CIDR removal.

Contrariwise, the administration of eCG or hCG after CIDR removal induced the appearance of estrus and fertile ovulations, with the eCG ewes displaying not only a higher estrus induction, but also an earlier onset of estrus with respect to the hCG ewes (*p* < 0.05; for both variables). However, the percentage of ewes ovulating and with corpus luteum on day 10 after CIDR removal was not different between the eCG ewes and the hCG ewes (*p* < 0.05). Besides, while the hCG ewes denoted an increased embryonic implantation rate, the eCG ewes displayed the largest pregnancy rate on day 32 after CIDR removal (*p* < 0.05).

**Figure 1:**
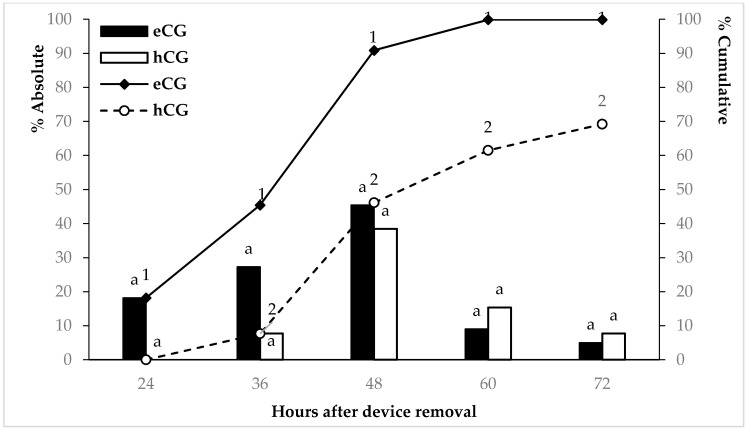
Absolute (bars) and cumulative (lines) percentages of Dorper sheep showing estrus signs over time after CIDR removal and administration of 300 IU of eCG or hCG (Groups eCG and hCG). a Different superscripts indicate significant differences in bars (*p* < 0.05); 1, 2 Different numbers indicate significant differences in lines (*p* < 0.05).

Regarding to the time to estrus onset (Figure 1), the eCG ewes showed the earliest average time for estrus manifestation after CIDR removal. Certainly, at 48 h after CIDR removal, around 90% of the females treated with eCG already presented estrus signs whilst only 49% of the females in the Group hCG showed such behavior. Finally, no differences in the remaining percentage of ewes showing estrus between the eCG and hCG ewes occurred from 48 to 72 h (*p* > 0.05) after CIDR removal.

**Figure 2:**
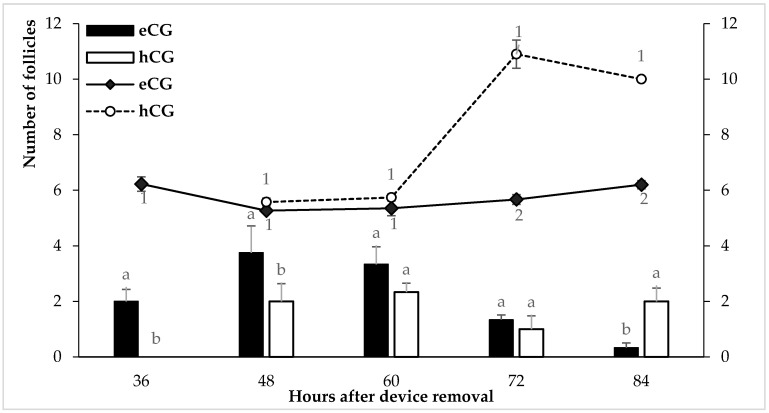
Mean diameter of the largest follicle (lines) and number of follicles ≥4 mm (bars) observed by transrectal ultrasonography after CIDR removal in Dorper sheep treated with 300 IU of eCG or hCG during seasonal anestrous. a, b Different superscripts indicate significant differences in bars (*p* < 0.05); 1, 2 Different numbers indicate significant differences in lines (*p* < 0.05).

These patterns of appearance of estrus induction were closely related to the patterns of follicle development observed during the preovulatory period (Figure 2). A higher number of ≥4 mm follicles was detected during the first 48 h after CIDR removal in the eCG ewes regarding the hCG ewes (*p* < 0.05); at time 36 h the eCG ewes showed a larger follicle diameter (*p* < 0.05). Conversely, the hCG ewes showed not only larger follicular diameters at 72 and 84 h but also a higher number of large follicles at 84 h after CIDR removal; this response was related to the presence of anovulatory follicular cysts. Concomitantly, the assessment of ovulatory events denoted no differences regarding the time to estrus onset after CIDR removal between the eCG and the hCG-treated ewes. On the other hand, while 100% ovulations on day 3 after CIDR removal occurred in the eCG ewes, only 33.5% of the hCG ewes ovulated on said day. However, by day 10 after CIDR removal, said ovulation differences vanished between eCG and hCG-treated ewes.

## 4. Discussion

The obtained results in the control ewes confirm that the Dorper multiparous females were in deep seasonal anestrous. The administration of hCG at the time of CIDR removal in sheep at seasonal anestrous was able to induce the resumption of the ovulatory activity in a narrow window of time. With respect to the eCG ewes, our results are in agreement with diverse studies (revised by Abecia et al. [1] and Gonzalez-Bulnes et al. [4]), which confirm that the eCG-treated ewes showed highly-synchronized estrus signs and fertile ovulations. Our results are in line with Ungerfeld and Rubianes, [23] and support all of the previous evidence on the benefits of applying eCG during seasonal anestrous for a synchronized resumption of ovulatory activity.

The results obtained in the control group showing no signs of estrus after being treated with exogenous progesterone, but without gonadotrophin stimulation, which should be related to the deficiencies in the final growth of ovulatory follicles during anestrous season previously reported [18]. There was, however, the presence of ovulation in around half of the control ewes on day 10 after CIDR removal. Such a scenario suggests that exposure to progesterone and active males was able to trigger the resumption of ovulatory response, although only in some of the animals (and in an extended period of time). Moreover, the results observed in the hCG ewes showed a lower synchronized activity when inducing estrus, ovulations and pregnancies as compared to the eCG ewes, the last in agreement with previous studies [17,24]. Hence, in view of these performances, the hCG treatment would not be as useful as the eCG administration when implementing artificial insemination protocols.

The assessment of the ovarian dynamics indicates that more than a half of the animals showing estrus signs in the hCG ewes evidenced failures in the ovulatory process on day 3 due to the presence of anovulatory follicular cysts. One possible explanation is that the low LH pulsatility during anestrous is not enough to trigger the final maturation of the preovulatory follicle and, therefore, the estradiol secretion from such ovarian follicle is not the required to modulate changes in FSH secretion from the pituitary [25] because the growing follicles need FSH to continue their development [26]. Such needs may have been fulfilled in those ewes treated with eCG, due to its biological action similar to FSH, while hCG only has similarity to LH [27], which precludes any effect on previous FSH-related follicle development. Moreover, the half-life of hCG is longer than 36 h in humans and 39 h in goats [28,29], so a single injection of hCG mimics the LH surge but remains in the blood beyond the normal duration of the LH surge, which may be the cause of the anovulatory luteinized follicles [30].

In spite of the lack of synchronized ovulations during the first 72 h after CIDR removal, around 85% of the hCG ewes denoted presence of ovulations on day 10 after progesterone removal, which may indicate the usefulness of hCG for inducing the resumption of the ovulatory activity of anestrous sheep, although in a narrow window of time. Our proposed treatment, based on the administration of hCG at the timing of progesterone removal, simplifies the management of the animals more than previous treatments including the administration of hCG at 24 h after CIDR removal and, therefore, one more handling day [17]. Therefore, if males remain with females for some days, it can be adopted by practitioners aiming to induce pregnancies in a short period of time. Interestingly, during the follow-up of the follicular growth waves, cyst structures were observed in the hCG ewes, suggesting that, irrespectively that these females depicted a delayed ovulation, a longer time is required to better follow-up the follicular wave. This issue deserves to be elucidated in future studies.

## 5. Conclusions

As expected, while the control ewes confirmed that the Dorper multiparous females were in deep seasonal anestrous, the eCG ewes showed a highly synchronized estrus manifestation and fertile ovulations. Regarding the administration of hCG at the time of CIDR removal in sheep during the anestrous season, it showed to be effective to induce estrus, promote follicular growth and a delayed yet significant ovulation (>84%) on day 10 after CIDR removal; furthermore, an increased embryonic implantation rate was also observed. Moreover, if the hCG ewes remain exposed to active males for some days, said strategy could be adopted, aiming to induce pregnancies by natural mating in a short period of time.

## Figures and Tables

**Table 1:** Reproductive outcomes of Dorper ewes treated with CIDR during the seasonal anestrous and, at the time of CIDR removal, receiving either 300 IU of eCG or hCG (groups eCG and hCG) or no hormones (group CON).

Response Variables	eCG	hCG	CON
Estrus induction (%)	100; (11/11) ^a^	69.3; (9/13) ^b^	0; (0/12) ^c^
Time CIDR removal to estrus (h)	41.45 ± 3.38 ^b^	52 ± 2.88 ^a^	-
Ovulations at day 3 (%)	100; (11/11) ^a^	38.5; (5/13) ^b^	0; (0/12) ^c^
Timing to ovulation (h)	70.91 ± 3.01 ^a^	74.40 ± 1.48 ^a^	-
Ovulations at day 10 (%)	100; (11/11) ^a^	84.6; (11/13) ^a^	50; (6/12) ^b^
Ovulation rate	1.91 ± 0.25 ^a^	2.0 ± 0.14 ^a^	1.16 ± 0.011 ^b^
Embryo implantation rate	1.63 ± 0.16 ^b^	2.0 ± 0.11 ^a^	0 ^c^
Pregnancy rate (%)	72.7; (8/11) ^a^	30.7; (4/13) ^b^	0; (0/12) ^c^

^a,b^ Different subscripts within response variable denote differences among groups (*p* < 0.05).

## Data Availability

Data are contained within the article.

## References

[1] Abecia J.A., Forcada F., Gonzalez-Bulnes A. (2012). Hormonal control of reproduction in small ruminants. Anim. Reprod. Sci..

[2] Murphy B.D. (2018). Equine chorionic gonadotropin: An enigmatic but essential tool. Anim. Reprod..

[3] Manteca-Vilanova X., De Briyne N., Beaver B., Turner P.V. (2019). Horse welfare during equine Chorionic Gonadotropin (eCG) production. Animals.

[4] Gonzalez-Bulnes A., Menchaca A., Martin G.B., Martinez-Ros P. (2020). Seventy years progestagen treatments for management of the sheep oestrous cycle: Where we are and where we should go. Reprod. Fertil. Dev..

[5] Lee S.Y., Byambaragchaa M., Kim J., Seong H., Kang M., Min K.S. (2017). Biochemical Characterization of Recombinant Equine Chorionic Gonadotropin (rec-eCG), using CHO Cells and PathHunter Parental Cells Expressing Equine Luteinizing Hormone/Chorionic Gonadotropin Receptors (eLH/CGR). J. Life Sci..

[6] Byambaragchaa M., Lee S.Y., Kim D.J., Kang M.H., Min K.S. (2018). Signal Transduction of Eel Luteinizing Hormone Receptor (eelLHR) and Follicle Stimulating Hormone Receptor (eelFSHR) by Recombinant Equine Chorionic Gonadotropin (rec-eCG) and Native eCG. Dev. Reprod..

[7] Min K.S., Park J.J., Byambaragchaa M., Kang M.H. (2019). Characterization of tethered equine chorionic gonadotropin and its deglycosylated mutants by ovulation stimulation in mice. BMC Biotechnol..

[8] Crispo M., Meikle M.N., Schlapp G., Menchaca A. (2021). Ovarian superstimulatory response and embryo development using a new recombinant glycoprotein with eCG-like activity in mice. Theriogenology.

[9] Martinez-Ros P., Gonzalez-Bulnes A. (2019). Efficiency of CIDR-based protocols including GnRH instead of eCG for estrus synchronization in sheep. Animals.

[10] Santos-Jimenez Z., Guillen-Gargallo S., Encinas T., Berlinguer F., Veliz-Deras F.G., Martinez-Ros P., Gonzalez-Bulnes A. (2020). Use of propylene-glycol as a cosolvent for GnRH in synchronization of estrus and ovulation in sheep. Animals.

[11] Santos-Jimenez Z., Martinez-Herrero C., Encinas T., Martinez-Ros P., Gonzalez-Bulnes A. (2020). Comparative efficiency of oestrus synchronization in sheep with progesterone/eCG and progesterone/GnRH during breeding and non-breeding season. Reprod. Domest. Anim..

[12] Hafez E.S.E., Jainudeen M.R., Rosnina Y., Hafez B., Hafez E.S.E. (2000). Reproduction in Farm Animals.

[13] Lapthorn A.J., Harris D.C., Littlejohn A., Lustbader J.W., Canfield R.E., Machin K.J., Morgan F.J., Isaacs N.W. (1994). Crystal structure of human chorionic gonadotropin. Nature.

[14] Kinser A.R., Gibson M.F., Vincent D.L., Scheffrahn N.S., Kesler D.J. (1983). Ovarian responses of seasonally anoestrus ewes administered progesterone, PMS, hCG and(or) GnRH. Theriogenology.

[15] Zamiri M.J., Hosseini M. (1998). Effects of human chorionic gonadotropin (hCG) and phenobarbital on the reproductive performance of fat-tailed Ghezel ewes. Small Rumin. Res..

[16] Dias L.M.K., Sales J.N.S., Viau P., Barros M.B.P., Nicolau S.S., Simões L.M.S., Alves N.G., Alonso M.A., Valentim R., Oliveira C.A. (2018). Although it induces synchronized ovulation, hCG reduces the fertility of Santa Ines ewes submitted to TAI. Arq. Bras. Med. Veterinária Zootec..

[17] Bruno-Galarraga M., Cano-Moreno V., Lago-Cruz B., Encinas T., Gonzalez-Bulnes A., Martinez-Ros P. (2021). The Use of hCG for Inducing Ovulation in Sheep Estrus Synchronization Impairs Ovulatory Follicle Growth and Fertility. Animals.

[18] Bartlewski P.M., Beard A.P., Cook S.J., Rawlings N.C. (1998). Ovarian follicular dynamics during anoestrus in ewes. Reproduction.

[19] Rosa H.J.D., Bryant M.J. (2003). Seasonality of reproduction in sheep. Small Rumin. Res..

[20] FASS (2010). Guide for the Care and Use of Agricultural Animals in Agricultural Research and Teaching.

[21] NAM-National Academy of Medicine (2010). Co-Produced by the National Academy of Medicine–Mexico and the Association for Assessment and Accreditation of Laboratory Animal Care International. Guide for the Care and Use of Laboratory Animals.

[22] Veiga-Lopez A., Encinas T., McNeilly A.S., Gonzalez-Bulnes A. (2008). Timing of preovulatory LH surge and ovulation in superovulated sheep are affected by follicular status at start of the FSH treatment. Reprod. Domest. Anim..

[23] Ungerfeld R., Rubianes E. (2002). Short term primings with different progestogen intravaginal devices (MAP, FGA and CIDR) for eCG-estrous induction in anoestrus ewes. Small Rumin. Res..

[24] Dias J.H., Miranda V.O., Oliveira F.C., Junior S.V., Haas C.S., Costa V.G.G., Lucia T., Vieira A.D., Corcini C.D., Gasperin B.G. (2020). Treatment with eCG and hCG to induce onset of estrous cycles in ewes during the non-breeding season: Effects on follicular development and fertility. Anim. Reprod. Sci..

[25] Souza C.J.H., Campbell B.K., Baird D.T. (1996). Follicular dynamics and ovarian steroid secretion in sheep during anoestrus. Reproduction.

[26] Bartlewski P.M., Beard A.P., Rawlings N.C. (1999). Ovarian function in ewes at the onset of the breeding season. Anim. Reprod. Sci..

[27] Ziecik A.J., Kaczmarek M.M., Blitek A., Kowalczyk A.E., Li X., Rahman N.A. (2007). Novel biological and possible applicable roles of LH/hCG receptor. Mol. Cell. Endocrinol..

[28] Duncan W.C., Kovacs G., Rutherford A., Gardne D.K. (2019). Physiology of Ovulation. How to Prepare the Egg and Embryo to Maximize IVF Success.

[29] Saleh M., Shahin M., Wuttke W., Gauly M., Holtz W. (2012). Pharmacokinetics of human chorionic gonadotropin after im administration in goats (Capra hircus). Reproduction.

[30] Parmar S.C. (2015). Anovulation, delayed ovulation and luteal insufficiency. Trends Biosci..

